# Evaluation of Condylar Positional, Structural, and Volumetric Status in Class III Orthognathic Surgery Patients

**DOI:** 10.3390/medicina56120672

**Published:** 2020-12-06

**Authors:** Jevgenija Podčernina, Ilga Urtāne, Pertti Pirttiniemi, Ģirts Šalms, Oskars Radziņš, Jolanta Aleksejūnienė

**Affiliations:** 1 Department of Orthodontics, Rīga Stradiņš University, Dzirciema str. 20, LV-1007 Rīga, Latvia; ilga.urtane@stomatologijasinstituts.lv (I.U.); oskars.radzins@stomatologijasinstituts.lv (O.R.); 2 Department of Oral Development and Orthodontics, University of Oulu, 90014 Oulu, Finland; pertti.pirttiniemi@oulu.fi; 3 Medical Research Center (MRC), Oulu University Hospital, 90014 Oulu, Finland; 4 Department of Oral and Maxillofacial Surgery, Rīga Stardiņš University, Dzirciema str. 20, LV-1007 Rīga, Latvia; girts.salms@stomatologijasinstituts.lv; 5 Department of Oral Health Sciences, Faculty of Dentistry, The University of British Columbia, Vancouver, BC V6T1Z3, Canada; jolanta@dentistry.ubc.ca

**Keywords:** condylar position, condylar volume, Class III deformities, orthognathic surgery

## Abstract

Background and objectives: The need to evaluate the condylar remodeling after orthognathic surgery, using three-dimensional (3D) images and volume rendering techniques in skeletal Class III patients has been emphasized. The study examined condylar positional, structural, and volumetric changes after bimaxillary or single-jaw maxillary orthognathic surgeries in skeletal Class III patients using the cone-beam computed tomography. Materials and Methods: Presurgical, postsurgical, and one-year post-surgical full field of view (FOV) cone-beam computed tomography (CBCT) images of 44 patients with skeletal Class III deformities were obtained. Group 1 underwent a bimaxillary surgery (28 patients: 24 females and 4 males), with mean age at the time of surgery being 23.8 ± 6.0 years, and Group 2 underwent maxillary single-jaw surgery (16 patients: 8 females and 8 males), with mean age at the time of surgery being 23.7 ± 5.1 years. After the orthognathic surgery, the CBCT images of 88 condyles were evaluated to assess their displacement and radiological signs of bone degeneration. Three-dimensional (3D) condylar models were constructed and superimposed pre- and postoperatively to compare changes in condylar volume. Results: Condylar position was found to be immediately altered after surgery in the maxillary single-jaw surgery group, but at the one-year follow-up, the condyles returned to their pre-surgical position. There was no significant difference in condylar position when comparing between pre-surgery and one-year follow-up in any of the study groups. Condylar rotations in the axial and coronal planes were significant in the bimaxillary surgery group. No radiological signs of condylar bone degeneration were detected one year after the surgery. Changes in condylar volume after surgery were found to be insignificant in both study groups. Conclusions: At one year after orthognathic surgery, there were no significant changes in positional, structural, or volumetric statuses of condyles.

## 1. Introduction

Class III skeletal deformities are the most frequent types of malocclusion requiring correction by orthognathic surgery [1,2]. Correction of skeletal Class III dentofacial deformities can be accomplished by maxillary advancement, mandibular setback, or bimaxillary surgery. Bimaxillary surgery with a simultaneous maxillary advancement and mandibular setback has become a common procedure as it provides a satisfactory facial appearance [3], preserves the volume of the upper airways [4] and is considered to be a stable procedure [5,6]. 

Mandibular osteotomies with consequent relocation and fixation of bony segments for the correction of skeletal dentofacial deformities can lead to condylar displacement and rotation within the glenoid fossa [7,8]. Condylar positional changes may develop due to several reasons, including ineffective interosseous fixation [9,10], misalignment of bone fragments [11,12], or tension in the surrounding muscles [13]. Maxillary osteotomies may indirectly alter the position of the condyle within the glenoid fossa by causing the autorotation of the mandible [14,15]. Only a few previous studies reported on alterations of the condyle after the isolated maxillary surgery [16,17,18,19].

Altered condylar position in orthognathic surgery patients causes mechanical stress on the condylar surface, and subsequent condylar dimensional changes occur in the postoperative period as areas of bone apposition and resorption form. Depending on the extent to which bone is lost, condylar changes can be physiological, defined as condylar remodeling, or pathological—condylar resorption [20,21]. 

Altered functional load, which is due to orthognathic surgery-induced condylar displacement, can compromise condylar bone structure and, consequently, radiological signs of bone degeneration may be detected [22,23,24,25,26]. Degenerative joint disease (DJD) is a process of tissue deterioration in which soft tissue, cartilage, and bone are converted into or replaced by inferior quality tissue [23]. The normal osseous components of the temporomandibular joints exhibit smooth, rounded articular surfaces without evidence of subchondral defects. As DJD progresses, the onset of erosive lesions occurs in loaded areas, and late-stage changes include the formation of osteophytes and subchondral bone cysts [25].

Condylar changes following the orthognathic surgery were mainly studied in skeletal Class II patients. The necessity of further studies, which utilize 3D images, volume rendering techniques and volume calculations in skeletal Class III patients has been recommended [27]. Cone-beam computed tomography (CBCT) scans allow us to compute and superimpose three-dimensional (3D) reconstructions of the mandibular condyles [28,29,30,31]. Using this novel image modality, accurate quantitative analyses of postoperative condylar volumetric changes can be performed [32,33]. In addition, the CBCT provides multiplanar reformatted (MPR) images for accurate and reliable linear measurements and identification of condylar bone structural changes [26,34,35,36,37]. 

The current study utilized cone-beam computed tomography to evaluate condylar positional, structural, and volumetric changes that may have occurred after bimaxillary or maxillary single-jaw orthognathic surgeries in skeletal Class III patients.

## 2. Materials and Methods

The database of all patients who received orthognathic surgery treatments from 2009 to 2018 at the Department of Maxillofacial Surgery of Rīga Stradiņš University, Latvia, was reviewed. The inclusion criteria comprised patients with a skeletal Class III malocclusion (cephalometric parameter: angle formed between the A point, Nasion point and B point (ANB angle) less than 2°) and those who had undergone bimaxillary (one-piece Le Fort I osteotomy for maxillary advancement and bilateral sagittal split osteotomy (BSSO) for mandibular setback) or single-jaw (one-piece Le Fort I osteotomy for maxillary advancement only) orthognathic surgery. Patients with syndromes, facial trauma, cleft lip and palate, signs and symptoms of temporomandibular joint disorders, or severe facial asymmetry (i.e., menton deviations of more than 4 mm from the facial midline) were excluded. 

Subsequently, information about 69 eligible patients (138 condyles) was retrieved. CBCT images from after the surgery (T1) were missing for 11 patients in the bimaxillary surgery group and 14 patients in the single-jaw surgery group; full CBCT records were available for 44 patients (88 condyles). These patients were selected for the current study and comprised two study groups. The bimaxillary surgery group was composed of 28 patients (24 females and 4 males), and their mean age at the time of surgery was 23.8 ± 6.0 years. The maxillary single-jaw surgery group included 16 patients (8 females and 8 males), and their mean age at the time of surgery was 23.7 ± 5.1 years. During the BSSO procedure, the mandibular condyles were positioned using bi-vectoral seating method and fixed using a 2.0 mm miniplate and one positioning screw on each side. The mean amount of surgical movement for the maxillary advancement was 4.6 ± 1.0 mm and for the mandibular setback—4.8 ± 1.0 mm. The Le Fort I osteotomy was fixed using two 2.0 mm L-shaped miniplates on each side. The mean amount of maxillary advancement in single-jaw surgery group was 5.7 ± 1.4 mm. All patients underwent orthodontic treatment before and after surgery at the Department of Orthodontics of Rīga Stradiņš University, Latvia. The study was approved by the Ethical Committee of Rīga Stradiņš University (Ethical Committee Approval Nr. E-9(2), approval date: 27 Feb 2014), and written informed consent was obtained from all patients. 

The CBCT images were obtained before surgery (0.8 ± 0.9 months), after surgery (0.6 ± 0.7 months), and at follow-up (13.1 ± 2.6 months). An i-CAT New Generation (Imaging Sciences International, Inc. Hatfield, PA, USA) CBCT machine was used for this, with patients seated in an upright position, their head in a natural position and teeth in maximum intercuspation. A standardized protocol was used for the equipment (voltage 120 kV; current 38 mA; FOV 17 cm; resolution 0.4 voxels; an approximate radiation dose of 36 μSv). The CBCT images were processed and analyzed using OsiriX MD 10.0.1 software, Pixmeo, Switzerland.

Two examiners performed the measurements. The first examiner, J.P. (Orthodontist) took linear, angular measurements and performed the cephalometric analysis. The first examiner was trained and calibrated by an experienced maxillofacial radiologist (L.N.) to identify condylar bone structural changes. The standardization (level of agreement) concerning image interpretations was high (Cohen’s Kappa coefficient was 0.819). After the standardization, the first examiner evaluated all study sample twice, 2 weeks apart and a high level of the intra-examiner agreement was found (intraclass correlation coefficient was above 0.840 for numerical variables; Cohen’s Kappa was above 0.819 for categorical variables). The second examiner, the medical engineer was responsible for condylar three-dimensional reconstructions, superimpositions, and volume calculations. Moreover, the second examiner assessed volumes twice, with a 2-week interval in-between the duplicate assessments, the intraclass correlation coefficient was above 0.840, which indicates a high level of intra-examiner agreement. 

### 2.1. Condylar Displacement

To evaluate changes in the anteroposterior condylar position (Χ) in the glenoid fossa, the anterior and posterior interarticular spaces on sagittal CBCT MPR images were measured in millimeters. The anterior space (A) was the distance between the most anterior condylar point to the corresponding glenoid fossa bone, and the posterior space (P) was the distance between the most posterior condylar point to the corresponding glenoid fossa bone.

**Figure 1:**
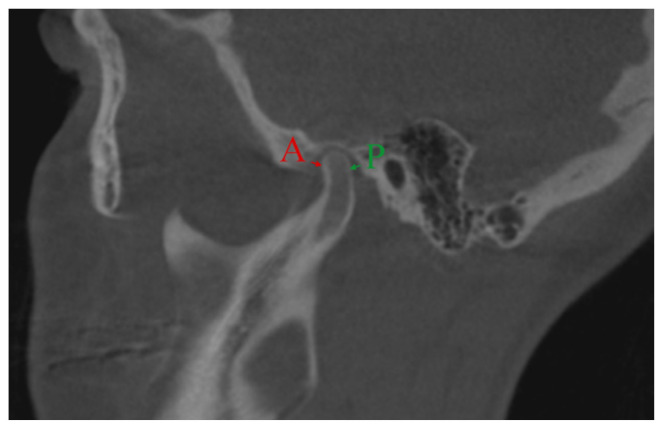
Linear measurements of the joint spaces on a sagittal cone-beam computed tomography (CBCT) multiplanar reformatted (MPR) image. Anterior (A) and posterior (P) joint spaces were measured between the most anterior and most posterior condylar head points to the glenoid fossa.

Standardized orientation for measurements in the sagittal plane was achieved by sectioning the CBCT image parallel to the line passing through the tip of the coronoid process and anterior margin of external auditory meatus as described by Hilgers et al. [35]. Measurements were analyzed using Pullinger and Hollender’s formula [38] (Figure 1). The condylar position (Χ) was determined as either concentric (−12% < Χ < 12%), anterior (Χ > 12%), or posterior (Χ < 12%).

### 2.2. Condylar Axial Changes

**Figure 2:**
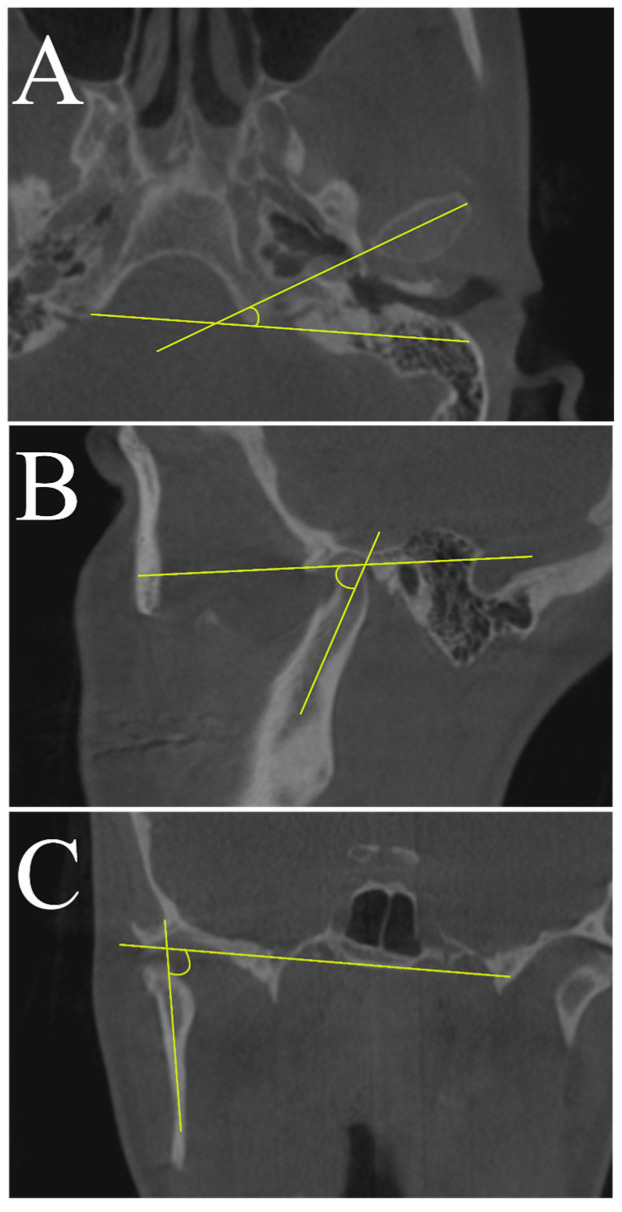
Illustration of the three measured angles: the axial condylar angle (**A**); the sagittal condylar angle (**B**); the coronal condylar angle (**C**).

To evaluate changes in the condylar axis, angular measurements between the reference planes and the axes (Table 1) were obtained on axial, sagittal, and coronal CBCT MPR images (Figure 2). 

On the axial view of the MPR image, the angle formed by the axial condylar axis and the coronal reference plane was defined as the axial plane angle (Figure 2A). On the coronal view of the MPR image, the angle between the coronal condylar axis and the Frankfurt horizontal plane, measured at the internal part of the intersection, was defined as the coronal plane angle (Figure 2B). On the sagittal view, the angle formed by the sagittal condylar axis and the Frankfurt plane was defined as the sagittal plane angle (Figure 2C). 

### 2.3. Condylar Bone Structural Changes 

Qualitative changes in the condylar bone structure were assessed on the sagittal and coronal CBCT MPR images following the radiographic criteria for condylar bone degeneration [25]. The condyles were evaluated for the presence or absence of the following radiographic features: 

Surface flattening—a loss of the rounded contour of the surface;

Subcortical sclerosis—an area of increased cortical bone density extending into the bone marrow;

Subcortical cyst—a cavity below the articular surface that deviates from the normal marrow pattern;

Surface erosion—an area of decreased density or discontinuity or irregularity in the cortical bone;

Osteophyte—marginal hypertrophy with sclerotic borders and exophytic angular formation of osseous tissue arising from the surface.

According to the detected radiographic structural changes, condyles were classified as normal, having condylar remodeling, or having active or stable degenerative joint disease.

### 2.4. Condylar Volume

The condylar module volumetric measurements were acquired using two software programs: 3D Slicer, Version 4.10.0 (open-source software, www.slicer.org) [39,40] to create surface models of the mandible and the cranial base accompanied by a module to enable model cutting and volume calculations, and Autodesk Meshmixer, Version 3.5 (open-source software, www.meshmixer.com) to trim the models to increase the computational speed and separate the cranial base from the mandible. After separation, each condylar region was exported as a separate model in stereolithography (STL) file format. Subsequently, these models were then imported back into the 3D Slicer software and cut using the EasyClip module, which provides the option of using up to three cutting planes.

Patients’ Digital Imaging and Communications in Medicine (DICOM) files were exported in a DICOM series format into the 3D Slicer software in which the segmentation tool was used for the creation of surface models using a semi-automatic approach. First, a lower threshold level of 130–250 HU (Hounsfield units), depending on the quality of the CBCT scan, was applied to maximize the signal-to-noise ratio and minimize manual work thereafter. Then, a manual approach was taken to edit any condylar boundary areas as deemed necessary. Lastly, the inside of each condylar surface model was filled just past the identified sigmoid notch using the island selection tool, thereby removing the effect of model wall thickness on the volume measurement. Once completed, the models were exported in STL format for further processing.

The exported STL models were then imported into the Autodesk Meshmixer software (Version 3.5). Here, each model was trimmed down to remove unnecessary data, including noise and artifacts, and to increase the computation time. For each patient, the mandibular regions containing the condyles and part of the cranial base were exported as separate new models in STL format.

**Figure 3:**
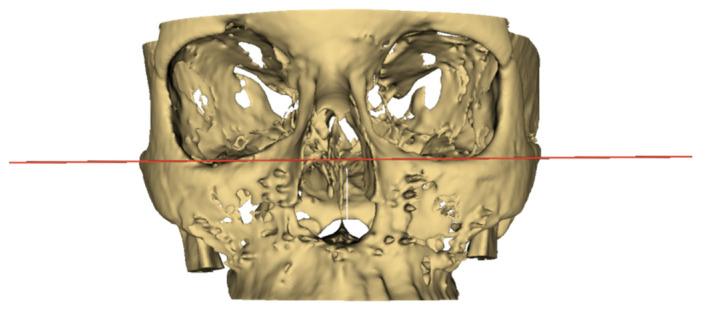
The position of the horizontal cutting plane.

**Figure 4:**
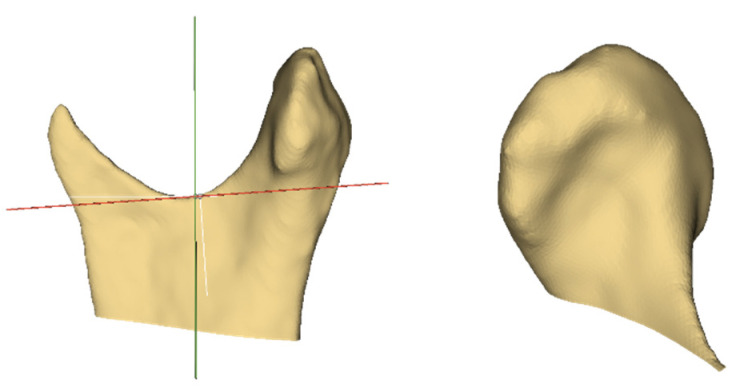
The left image shows the horizontal (red) and vertical (green) cutting planes in sagittal view for a single model. The right image shows the resultant model used for volumetric measurements.

These new models were then imported back into the 3D Slicer software, and the EasyClip module was applied. This is where the cranial base model was used as a reference to position the main cutting plane—in Figure 3, the red plane was positioned as the Frankfurt horizontal plane. Once set, the cranial base model was hidden, and the horizontal cutting plane was moved downwards without changing its angular position to go through the sigmoid notch of each mandibular model separately. The coronal cutting plane (indicated by the green line in Figure 4) was positioned to pass through this intersection between the cutting plane and the sigmoid notch in case the horizontal plane was positioned too low. The result for a single sample is exemplified in Figure 4. Once complete, each model’s information tab was opened to record the volume. 

### 2.5. Statistical Analyses 

IBM SPSS Statistics for Windows, Version 22.0 (IBM Corp, Armonk, NY, USA) software was used for all statistical analyses. The results were considered statistically significant when *p* < 0.05. 

Both, the condylar position relative to the glenoid fossa and the morphological status before the surgery, after the surgery, and at the subsequent follow-up were compared by the chi-square test for non-related comparisons or McNemar test for related comparisons, or Fisher’s exact test (when conditions for the chi-square test were not met). Condylar axis changes in each group were examined at three time-points, and time-related changes were analyzed using a paired samples *t*-test (for normally distributed data) or Mann–Whitney test (for non-normally distributed data). Condylar volume statuses between the surgery and after the surgery were compared using a paired samples *t*-test. An independent sample *t*-test was employed to compare mean changes in the condylar volume and axis between the two surgery groups. 

## 3. Results

CBCT images of 44 patients were included in the study. Demographic and clinical characteristics of the study sample are listed in Table 2. The positional, structural, and volumetric statuses of 88 condyles were assessed before and after orthognathic surgery. 

### 3.1. Condylar Positional Changes

#### 3.1.1. Bimaxillary Surgery Group

There were no time-related differences in any of the condylar position subgroups—concentrically (A), anteriorly (B), or posteriorly (C). At T0, there was a significantly lower proportion of posteriorly positioned condyles (C) as compared with the proportions of those positioned concentrically (A) (*p* = 0.020) or anteriorly (B) (*p* = 0.002). Similar patterns concerning variances among different condylar positions were observed at T1 and T2. The proportions of condylar positions are shown in Table 3. 

#### 3.1.2. Maxillary Single-Jaw Surgery Group

There were no significant time-related changes in either the concentric (A¹) or anterior (B¹) condylar position subgroups. In the posteriorly (C^1^) positioned condyles subgroup, there were significant changes between T0 and T1 (*p* = 0.043), T1 and T2 (*p* = 0.043), but not between T0 and T2 (*p* = 1.000). Significant variation among differently positioned condyles was observed at T0 and T2, but not at T1. A significantly lower proportion of posteriorly (C^1^) positioned condyles was detected at T0 as compared with the proportion of positioned concentrically (A^1^) (*p* = 0.001) or anteriorly (B^1^) (*p* = 0.001). Similar patterns concerning variances among different condylar positions were observed at T2. The proportions of condylar positions are shown in Table 3.

#### 3.1.3. Between-Group Comparisons

No statistically significant differences were found in the proportions of the same type of condylar positions between the bimaxillary and maxillary single-jaw surgery groups at either pre-surgery (T0) or post-surgery (T1 or T2) comparisons (Table 3).

Table 3 presents several types of comparisons regarding the condylar position before and after surgery. The horizontal time-related comparisons were completed for each condylar position-related subgroup and separately for the bimaxillary and maxillary single-jaw surgery groups. In both surgery groups, subgroup A consisted of condyles positioned concentrically, subgroup B consisted of condyles positioned anteriorly, and subgroup C consisted of the posteriorly positioned condyles. The following time-related comparisons were conducted: T0 with T1, T1 with T2, and T0 with T2 (e.g., proportion of condyles with the concentric position in the bimaxillary surgery group at T0 and T1). The vertical comparisons demonstrate differences among subgroups (A, B, and C) in the same surgery group at a specific observation time, that is, at T0, T1, or T2 (e.g., in the bimaxillary surgery group, the proportions of different types of condylar positions are compared at T0). The third type of analysis compared the proportions of specific condylar positions (A, B, and C) between the bimaxillary and maxillary single-jaw surgery groups at a specific time (T0, T1, or T2) (e.g., the proportion of condyles between the bimaxillary and maxillary single-jaw surgery groups with a concentric position at T0).

### 3.2. Changes in the Condylar Axis

#### 3.2.1. Bimaxillary Surgery Group

There was a significant increase in the axial plane angle, 3.0° on average (*p* ˂ 0.001; 95% CI 2.0; 4.) from T0 to T1, and 2.6° on average (95% CI 3.6; 1.5) from T0 to T2. There were no significant (*p* = 0.271) mean changes in the axial plane angles from T1 to T2. There was also a significant increase in the coronal plane angles, 2.1° on average (*p* ˂ 0.001; 95% CI –3.0; –1.1) from T0 to T1 and 1.3° on average (*p* = 0.007; 95% CI 2.2; 0.4) from T0 to T2. On average, from T1 to T2, the coronal plane angles significantly decreased by 0.8° (*p* = 0.017; 95% CI –0.2; –1.4). No significant mean changes in sagittal plane angles were found between T0 and T1 (*p* = 0.480), T1 and T2 (*p* = 0.676), or T0 and T2 (*p* = 0.271); see Table 4. 

#### 3.2.2. Maxillary Single-Jaw Surgery Group

No significant changes in the means of the axial plane angles occurred between T0 and T1 (*p* = 0.145), T1 and T2 (*p* = 0.241), or T0 and T2 (*p* = 0.615). A significant decrease, 0.4° on average (*p* = 0.012; 95% CI –0.1; –0.6), was found in the coronal plane angle from T1 to T2. No significant mean changes were observed for the coronal plane angles from T0 to T1 (*p* = 0.450) or from T0 to T2 (*p* = 0.116). There were no significant mean changes in the sagittal plane angles from T0 to T1 (*p* = 0.911), T1 to T2 (*p* = 0.235), or T0 to T2 (*p* = 0.229); see Table 4. 

#### 3.2.3. Between-Group Comparisons

The means of the condylar axial plane angles differed significantly (*p* = 0.005) between the two surgery groups at T1; the bimaxillary group angles were, on average, 4.1° higher than the maxillary single-jaw surgery group. The means of the condylar axial plane angles also differed significantly (*p* = 0.007) between the two surgery groups at T2; the bimaxillary group angles were, on average, 3.8° higher than the maxillary single-jaw surgery group. No significant mean differences in either the coronal or sagittal plane angles were observed between the maxillary single-jaw and bimaxillary surgery groups (Table 4).

Table 4 presents time-related comparisons in condylar angles separately for the bimaxillary and the maxillary single-jaw surgery groups. 

### 3.3. Condylar Bone Structural Alterations

No significant changes in terms of condylar bone structural alterations were found in any of the pre- or post-surgery comparisons, neither in the bimaxillary group nor in the maxillary single-jaw surgery group. In fact, there were no significant differences before or after surgery between the two groups for any type of structural alteration (Table 5).

Table 5 compares several aspects of condylar bone structural alterations before and one year after surgery; the bimaxillary and maxillary single-jaw surgery groups were compared separately.

There were no statistically significant changes in the condylar status before or after surgery in either the bimaxillary or maxillary single-jaw surgery groups (Table 6). 

Table 6 presents the condylar status, before and after surgery, based on the radiologically detected condylar bone structural alterations; the bimaxillary and maxillary single-jaw surgery groups were analyzed separately.

### 3.4. Changes in Condylar Volume

No significant mean changes were found between the pre- and post-surgery condylar volumes in either the bimaxillary or maxillary single-jaw surgery groups. Furthermore, there were no significant mean differences between the bimaxillary and maxillary single-jaw surgery groups before or after jaw surgery (Table 7).

Table 7 presents the condylar volume-related within-group and between-group comparisons for the bimaxillary and the maxillary single-jaw surgery groups before and one year after the surgery

## 4. Discussion

Orthognathic surgery-induced changes in the condylar position may often result in condylar remodeling [41,42]. The current study utilized 3D CBCT images to assess condylar displacement, axial changes, and structural and volumetric changes after bimaxillary and maxillary single-jaw orthognathic surgeries in skeletal Class III patients. 

In the present study, the condylar displacements were not significantly different between the maxillary single-jaw and bimaxillary surgery groups at pre-surgical, post-surgical, or one-year follow-up time points. In the bimaxillary surgery group, the condyles had anterior or concentric positions in the glenoid fossa before surgery. After surgery, the pattern of condylar locations remained the same. At the one-year follow-up, the condyles tended to move more concentrically as compared with before or immediately after surgery. The proportions of anteriorly positioned condyles decreased only minimally. This finding is in accordance with the previous research of Kim et al. [43,44], Iguchi et al. [45], and Choi et al. [46,47]. In the maxillary single-jaw surgery group, the condyles were positioned anteriorly or concentrically before the surgery. The proportion of posteriorly positioned condyles significantly increased after surgery; however, one year after surgery, the condyles had moved to their original pre-surgical position. There are several reasons why condylar displacement may develop after maxillary single-jaw surgery. The mandibular autorotation that ensues from superior maxillary repositioning results in rotation of the condyle along its long axis, which may subsequently result in posterior displacement of the condyle as it rotates in the fossa. Soft tissue tension along with resistance within the maxillomandibular complex and a new occlusal position may also change the relationship between the condyle and the fossa [14,15]. 

To the best of our knowledge, only a few studies have evaluated condylar positional changes after maxillary single-jaw surgery for correction of dentofacial deformities. Cevidanes et al. [19] evaluated one-week post-surgery changes in condylar position using CBCT images of patients with different types of dentofacial deformities. Three-dimensional mandibular condyles and rami models were constructed from CBCT images and superimposed on the cranial base. Color maps were analyzed to assess the 3D condylar displacement. Only one out of 10 patients showed posterior mandibular rami displacement of more than 2 mm. Cortez et al. [18] studied patients who had skeletal Class III orthognathic surgery and used submentovertex radiographs to assess condylar positional changes after maxillary advancement surgery; no changes were observed in condylar position from pre- to post-surgery (1–2 weeks and six months after surgery). Mavreas and coworkers [17] studied tomograms of temporomandibular joints. Out of the 44 patients included in the study, 10 patients underwent isolated maxillary surgery for the correction of different types of dentofacial deformities. Posterior and anterior temporomandibular joint spaces were measured to assess condylar displacement before and 1 week and 6 months after orthognathic surgery. In that study, no changes in condylar position were found after the surgery.

In the current study, angular measurements in the axial, coronal, and sagittal planes were performed to assess condylar axial changes. A significant difference between the two groups was found for the condylar angular measurements in the axial plane when comparing post-surgery and one-year follow-up measurements. The bimaxillary surgery group showed, on average, a 4.0° higher inward rotation of the condylar head as compared with the maxillary single-jaw surgery group. 

In the bimaxillary surgery group, the condyles showed significant inward rotation immediately after surgery. The one-year follow-up axial plane angle values were at the same level as immediately after the surgery. This finding is consistent with previous studies [42,43,44,48]. The available evidence regarding angular rotation in the axial plane is controversial. Several studies [46,47,49,50] found that condyles tend to rotate inwards immediately after surgery. However, longer-term follow-ups showed that condyles tend to regress to their original position. In contrast to our findings, a study by Ueki et al. [51] found that condyles overcome outward rotation after surgery. 

The coronal plane angle significantly increased after surgery, indicating mesial tilting of the condyles. However, one year later, the condyles tended to realign. This is in accordance with the findings from Choi et al. [46]. Contrary to our findings, Ueki et al. [51] reported lateral tilting in the coronal plane 1 year after orthognathic surgery. No change in the coronal plane angle was reported by Kim et al. [43,44]. 

In the sagittal plane, the angle between the condylar long axis and the Frankfort plane decreased after surgery and at the one-year follow-up; this indicates that condyles tend to have backward rotation. Our findings are in line with the findings of Kim et al. [44] and Ueki et al. [51,52]. The Ueki et al. study [51] reported backward condylar rotation 1 week after surgery; however, one year after surgery, the condylar sagittal angle regressed to pre-surgery status. By contrast, Kim et al. [43] reported forward condylar rotation in the sagittal plane three months after orthognathic surgery. 

Differences in findings could be broadly explained by factors such as differences in condylar reposition methods, types of internal fixation, amount and direction of surgical movement, intermaxillary immobilization period, and their multiple interactions [53].

In the maxillary single-jaw surgery group, the only significant change in the coronal plane angle from the post-surgical to the one-year follow-up observation was found. However, the condylar coronal plane angle decrease on average by 0.4° is lacking clinical significance. Sanroman et al. [16] and Cortez et al. [18] studied condylar rotational displacement after the maxillary single-jaw surgery in patients with different types of malocclusions and they did not find condylar angular changes six months after the maxillary advancement orthognathic surgery. The absence of the condylar angular changes after the isolated maxillary advancement surgery could be explained by not substantial degree of maxillary repositioning to cause mandibular autorotation and masticatory muscles ability to adapt to a new environment. 

No significant condylar morphology differences were found between the bimaxillary and maxillary single-jaw surgery groups either before or after surgery. In the bimaxillary surgery group, the most common radiographic findings before and after surgery were condylar surface flattening and subcortical sclerosis. Before surgery, the majority of condyles (83.9%) were classified under the remodeling status, and a similar proportion (82.9%) was observed one year after surgery. One condyle (1.8%) was classified under the stable degenerative joint disease status due to the presence of a subcortical cyst. However, the subcortical cyst was detected before orthognathic surgery and did not show any further development at the one-year follow-up. 

In the maxillary single-jaw surgery group, condylar surface flattening was found in 26 (81.3%) and subcortical sclerosis in 2 (6.3%) out of the total 32 condyles. Increased frequency (12.5%; four new cases) of subcortical sclerosis was found 1 year after surgery. In general, most condyles had remodeling status both before (81.3%) and after (87.5%) orthognathic surgery. No features of condylar bone degeneration were observed in the maxillary single-jaw surgery group before or after surgery.

Little is known about the time-related condylar bone structural changes in patients who undergo orthognathic surgery for correction of skeletal Class III dentofacial deformities. Krisjane et al. [54] evaluated the prevalence of condylar bone structural changes in skeletal Class I, Class II, and Class III patients who sought combined orthodontic and orthognathic surgery treatments. The most common radiographic finding in the skeletal Class III group was articular surface flattening (47.0%; 23 out of 49 condyles) followed by osteophyte formation (20.4%; 10 out of 49 condyles), and surface erosion (12.3%; 6 out of 49 condyles). Subcortical sclerosis (2.0%; 1 out of 49 condyles) and subcortical cyst formation (2.0%; 1 out of 49 condyles) were not prevalent. The post-surgical evaluation was not included in this study. The cross-sectional study of Yamada et al. [55] assessed the pre-treatment helical CT images of 129 orthognathic surgery patients for the presence of condylar surface flattening, surface erosion, and osteophytes. Subcortical sclerosis or cysts were not observed in this study. Researchers concluded that the condylar bone changes were more frequent in mandibular retrusion and open bite cases. In the mandibular protrusion group, surface flattening was observed in 3.3% (3 out of 92 joints) and erosion in 2.2% (2 out of 92 joints) of all cases. In the maxillary retrusion group, the corresponding values were 8.3% (2 out of 24 joints) and 4.2% (1 out of 24 joints). No condylar bone structural changes were detected in the maxillary retrusion and mandibular protrusion groups. However, information about the post-surgery changes is not available. 

Since mandibular condyles demonstrate adaptability to changed functional stimuli after the orthognathic surgery, progressive changes with a decrease in condylar volume may alter skeletal stability post-surgery. The extent of condylar volume decrease is an important factor to consider. A study by Xi et al. [56] showed that loss of more than 17% of the original condylar volume is an indicator of pathological condylar resorption. In our study, condylar volume assessments showed no significant difference between the maxillary single-jaw and bimaxillary surgery groups for both the pre- and post-surgical mean values. Our study showed that in the bimaxillary surgery group, the condylar volume decreased, on average, by 9.2 ± 8 mm^3^ one year after surgery, which represents 5.3% of the pre-operative volume. In the maxillary single-jaw surgery group at one year after surgery, condylar volume increased, on average, by 16.7 ± 1.9 mm^3^, which represents 9.1% of the pre-operative volume. Lin et al. [57] assessed condylar volumetric changes in skeletal Class III patients with an asymmetric mandibular prognathism. In the BSSO group, the average volume changes of the deviated and non-deviated sides of the condyles were 71.6 ± 11.9 and 59.3 ± 8.4 mm^3^, respectively, one year after surgery. However, no data about the percentage decrease are available for the present study.

It is important to mention the study limitations. The scope of our study was to assess possible condylar changes in the context of different aspects such as position, structure, volume in the same sample at different time points. As clinical evaluation of the temporomandibular joint function was not the scope of the present study, our findings have limited clinical implications. We could speculate, that the absence of significant positional, structural or volumetric condylar changes indicates the absence of clinical signs and symptoms. However, these assumptions would require additional clinical evaluation which could be the focus of future studies. Our study groups were not homogenous, and the sample size of the maxillary single-jaw surgery group was relatively small; therefore, extrapolation of the present findings needs to be practiced with caution in terms of broader applicability. Due to the heterogeneity of the study groups we did not compare condylar changes with the degree of surgical movement. The standardization of the cross-sections in the assessment of multiplanar images for linear and angular measurements is challenging in longitudinal and across-subject studies. To mitigate this challenge, our examiner was trained and calibrated prior to the study. Furthermore, our assessment of condylar volumetric changes did not allow delimitation of the areas of bone resorption or apposition, nor assessment of whether these alterations were due to the repositioning of condyles. The use of color maps might be necessary in future research.

## 5. Conclusions

Our study suggests that condyles of skeletal Class III patients undergoing bimaxillary and maxillary single-jaw surgeries may experience marginal anteroposterior and axial displacement within the glenoid fossa, minor alterations of condylar bone structure, and minor volumetric changes.

## Figures and Tables

**Table 1:** Reference planes and axes.

Reference Planes/Axes	Description
Frankfurt horizontal plane	The plane passing through the right portion and the right and left orbits
Coronal plane	The plane perpendicular to the Frankfurt and midsagittal planes and passing through the basion point
Midsagittal plane	The plane perpendicular to the Frankfurt plane and passing through the nasion and basion points
Axial condylar axis	The axis passing through the most lateral and medial condylar points
Coronal condylar axis	The axis passing the center of the ramus between the lateral and medial condylar poles
Sagittal condylar axis	The axis passing the center of the condylar neck to the center of the condylar head

**Table 2:** Demographic data and clinical characteristics of the study sample *.

Variables	Bimaxillary Surgery	Maxillary Single-Jaw Surgery	Significance ^
	*N* (%)	*N* (%)	
Total Patients/Condyles *	28/56 (100.0)	16/32 (100.0)	
Gender: Females	24 (85.7)	8 (50.0)	<0.001
Males	4 (14.3)	8 (50.0)	
	**Mean (sd)**	**Mean (sd)**	
Age at surgery (in years) #	23.8 (6.0)	23.7 (5.1)	0.834
**Radiological Examinations** #			
Time of CBCT (in months)	**Mean (sd)**	**Mean (sd)**	
Before the surgery (T0)	0.8 (1.0)	0.8 (0.7)	0.526
After the surgery (T1)	0.7 (0.8)	0.5 (0.6)	0.266
One-year follow-up (T2)	12.6 (2.0)	13.9 (3.4)	0.491
**Cephalometric Characteristics:** angular measurements (°) #			
	**Mean (sd)**	**Mean (sd)**	
SNA angle	79.9 (3.5)	79.2 (3.5)	0.690
SNB angle	84.0 (4.4)	82.4 (4.5)	0.102
ANB angle	−4.1 (2.9)	−3.1 (2.2)	0.211
MP-SN angle	35.9 (5.6)	33.1 (7.8)	0.036
**Cephalometric Characteristics:** linear measurements (mm) #			
	**Mean (sd)**	**Mean (sd)**	
Witt’s appraisal	−11.2 (4.1)	−8.0 (3.0)	0.008
A to NP	−1.1 (4.5)	−3.2 (4.0)	0.120
Pog to NP	9.4 (6.3)	4.3 (6.2)	0.014

^—Chi-square test; #—Independent sample *t*-test (normal data) or Mann–Whitney test (non-normal data); *—Unit of analyses: condyles; CBCT—Cone-beam computed tomography; Abbreviations: SNA—angle measuring anteroposterior relationship of maxillary basal arch on anterior cranial base; SNB—angle measuring anteroposterior relationship of the mandibular basal arch on anterior cranial base; ANB—angle indicating anteroposterior relationship between the maxillary and mandibular basal arches; MP-SN—mandibular plane angle; A—the point of the deepest concavity on the anterior maxillary alveolus; Pog—the most anterior point of the mandibular symphysis; NP—nasion perpendicular.

**Table 3:** Condylar positional statuses before and after surgery.

Condylar Positions	T0	T1	T2	Significance ^		
	*N* (%)	*N* (%)	*N* (%)			
**Bimaxillary Surgery Group (*N* = 56)**				**T0-T1**	**T1-T2**	**T0-T2**
Concentric (A)	21 (37.5)	23 (41.1)	26 (46.4)	0.699	0.568	0.338
Anterior (B)	25 (44.6)	22 (39.3)	21 (37.5)	0.566	0.846	0.846
Posterior (C)	10 (17.9)	11 (19.6)	9 (16.1)	0.809	0.622	0.801
**Significance #**	**T0**	**T1**	**T2**			
A vs. B	0.442	0.847	0.338			
A vs. C	0.020	0.014	0.001			
B vs. C	0.002	0.023	0.010			
**Maxillary Single-Jaw Surgery Group (*N* = 32)**				**Significance ^**		
	**T0**	**T1**	**T2**	**T0-T1**	**T1-T2**	**T0-T2**
Concentric (A¹)	15 (46.9)	11 (34.4)	16 (50.0)	0.309	0.206	0.802
Anterior (B¹)	15 (46.9)	12 (37.5)	14 (43.8)	0.448	0.611	0.802
Posterior (C¹)	2 (6.3)	9 (28.1)	2 (6.3)	0.043	0.043	1.000
**Significance #**	**T0**	**T1**	**T2**			
A¹ vs. B¹	1.000	0.794	0.616			
A¹ vs. C¹	0.001	0.590	0.001			
B¹ vs. C¹	0.001	0.424	0.001			
**Bimaxillary Surgery VS. Maxillary Single-Jaw Surgery**						
Significance #	**T0**	**T1**	**T2**			
Concentric (A)	0.390	0.535	0.747			
Anterior (B)	0.840	0.869	0.564			
Posterior (C)	0.198	0.361	0.315			

#—Chi-square test; ^—McNemar test/Fisher’s exact test (when conditions for chi-square test were not met); T0—before surgery; T1—after surgery; T2—1 year after surgery.

**Table 4:** Condylar angular status in the bimaxillary and maxillary single-jaw surgery groups (before and after surgery).

Condylar Angles	T0	T1	T2	Significance ^		
	Mean (sd)°	Mean (sd)°	Mean (sd)°			
**Bimaxillary Surgery Group (*N* = 56)**				**T0-T1**	**T1-T2**	**T0-T2**
Axial plane	19.6 (7.5)	22.6 (7.9)	22.2 (7.6)	0.001	0.271	0.001
Coronal plane	83.8 (3.4)	85.9 (4.8)	85.1 (5.1)	0.001	0.017	0.007
Sagittal plane	59.3 (5.9)	58.9 (5.9)	58.8 (5.9)	0.480	0.676	0.271
**Maxillary Single-Jaw Surgery Group (*N* = 32)**				**Significance ^**		
	**T0**	**T1**	**T2**	**T0-T1**	**T1-T2**	**T0-T2**
Axial plane	18.3 (5.4)	18.5 (5.3)	18.4 (5.3)	0.145	0.241	0.615
Coronal plane	84.7 (3.2)	84.8 (3.3)	84.4 (3.2)	0.450	0.012	0.116
Sagittal plane	59.6 (4.6)	59.6 (4.5)	59.4 (4.7)	0.911	0.235	0.229
**Bimaxillary Surgery vs. Maxillary Single-Jaw Surgery**						
**Significance #**	**T0**	**T1**	**T2**			
Axial plane	0.333	0.005	0.007			
Coronal plane	0.243	0.256	0.452			
Sagittal plane	0.783	0.565	0.575			

^—Paired sample *t*-test; #—Independent sample *t*-test or Mann–Whitney test (non-normal data); T0—before the surgery; T1—after the surgery; T2—one year after the surgery.

**Table 5:** Comparison of condylar bone structural alterations in bimaxillary and maxillary single-jaw surgery groups (before and 1 year after surgery).

Alterations	T0	T2	Significance #
	*N* (%)	*N* (%)	
**Bimaxillary Surgery Group (*N* = 56)**			**T0-T2**
Surface flattening	48 (85.7)	50 (89.3)	0.568
Subcortical sclerosis	7 (12.5)	12 (21.4)	0.208
Subcortical cyst	1 (1.8)	1 (1.8)	1.000
Surface erosion	0 (0.0)	0 (0.0)	N/A
Osteophyte	0 (0.0)	0 (0.0)	N/A
**Maxillary Single-Jaw Surgery Group (*N* = 32)**			**Significance #**
	**T0**	**T2**	**T0-T2**
Surface flattening	26 (81.3)	26 (81.3)	1.000
Subcortical sclerosis	2 (6.3)	6 (18.8)	0.267
Subcortical cyst	0 (0.0)	0 (0.0)	N/A
Surface erosion	0 (0.0)	0 (0.0)	N/A
Osteophyte	0 (0.0)	0 (0.0)	N/A
**Bimaxillary Surgery vs. Maxillary Single-Jaw Surgery**			
**Significance #**	**T0**	**T2**	
Surface flattening	0.582	0.291	
Subcortical sclerosis	0.478	0.764	
Subcortical cyst	1.000	1.000	
Surface erosion	N/A	N/A	
Osteophyte	N/A	N/A	

#—Chi-square test or Fisher’s exact test (when conditions for chi-square test were not met). T0—before the surgery; T2—1 year after surgery; Abbreviations: N/A—not applicable.

**Table 6:** Condylar status in bimaxillary and maxillary single-jaw surgery groups according to radiologically detected signs of condylar bone structural alterations (before and 1 year after surgery).

Condylar Status	T0	T2	Significance #
	*N* (%)	*N* (%)	
**Bimaxillary Surgery Group (*N* = 56)**			**T0-T2**
Normal condyle	8 (14.3)	6 (10.7)	0.568
Remodeling	47 (83.9)	49 (87.5)	0.589
Active DJD	0 (0.0)	0 (0.0)	N/A
Stable DJD	1 (1.8)	1 (1.8)	1.000
**Maxillary Single-Jaw Surgery Group (*N* = 32)**			**Significance #**
	**T0**	**T2**	**T0-T2**
Normal condyle	6 (18.8)	4 (12.5)	0.732
Remodeling	26 (81.3)	28 (87.5)	0.732
Active DJD	0 (0.0)	0 (0.0)	N/A
Stable DJD	0 (0.0)	0 (0.0)	N/A
**Bimaxillary Surgery vs. Maxillary Single-Jaw Surgery**			
**Significance #**	**T0**	**T2**	
Normal condyle	0.582	1.000	
Remodeling	0.748	1.000	
Active DJD	N/A	N/A	
Stable DJD	1.000	1.000	

#—Chi-square test or Fisher’s exact test (when conditions for chi-square test were not met); T0—before surgery; T2—one year after surgery; Abbreviations: DJD—degenerative joint disease; N/A—not applicable.

**Table 7:** The condylar volumetric status in bimaxillary and maxillary single-jaw surgery groups (before and 1 year after surgery).

Condylar Volume	T0	T2	
	Mean (sd) mm³	Mean (sd) mm³	
**Bimaxillary Surgery Group (*N* = 56)**			**Significance ^**
	1738.1 ± 449.9	1728.9 ± 441.9	0.354
**Maxillary Single-Jaw Surgery Group (*N* = 32)**			
	**T0**	**T2**	
	1827.7 ± 534.0	1844.4 ± 532.1	0.105
**Bimaxillary Surgery vs. Maxillary Single-Jaw Surgery**			
**Significance #**	**T0**	**T2**	
	0.404	0.277	

^—Paired samples *t*-test; #—Independent sample *t*-test; T0—before the surgery; T2—one year after the surgery.
